# Sequencing and characterization of the complete mitochondrial genome of Japanese Swellshark (*Cephalloscyllium umbratile*)

**DOI:** 10.1038/s41598-017-15702-0

**Published:** 2017-11-10

**Authors:** Ke-Cheng Zhu, Yin-Yin Liang, Na Wu, Hua-Yang Guo, Nan Zhang, Shi-Gui Jiang, Dian-Chang Zhang

**Affiliations:** 1 Key Laboratory of South China Sea Fishery Resources Exploitation and Utilization, Ministry of Agriculture, South China Sea Fisheries Research Institute, Chinese Academy of Fishery Sciences, 231 Xingang Road West, Haizhu District, Guangzhou, 510300 China; 2 Engineer Technology Research Center of Marine Biological Seed of Guangdong Province, Guangzhou, Guangdong Province The People’s Republic of China; 3 Key Laboratory of Fishery Ecology & Environment, Guangdong Province, Guangzhou, Guangdong Province The People’s Republic of China; 4 South China Sea Bio-Resource Exploitation and Utilization Collaborative Innovation Center, Guangzhou, Guangdong Province The People’s Republic of China

## Abstract

To further comprehend the genome features of *Cephalloscyllium umbratile* (Carcharhiniformes), an endangered species, the complete mitochondrial DNA (mtDNA) was firstly sequenced and annotated. The full-length mtDNA of *C. umbratile* was 16,697 bp and contained ribosomal RNA (rRNA) genes, 13 protein-coding genes (PCGs), 23 transfer RNA (tRNA) genes, and a major non-coding control region. Each PCG was initiated by an authoritative ATN codon, except for *COX1* initiated by a GTG codon. Seven of 13 PCGs had a typical TAA termination codon, while others terminated with a single T or TA. Moreover, the relative synonymous codon usage of the 13 PCGs was consistent with that of other published Carcharhiniformes. All tRNA genes had typical clover-leaf secondary structures, except for tRNA-Ser (GCT), which lacked the dihydrouridine ‘DHU’ arm. Furthermore, the analysis of the average Ka/Ks in the 13 PCGs of three Carcharhiniformes species indicated a strong purifying selection within this group. In addition, phylogenetic analysis revealed that *C. umbratile* was closely related to *Glyphis glyphis* and *Glyphis garricki*. Our data supply a useful resource for further studies on genetic diversity and population structure of *C. umbratile*.

## Introduction

*Cephalloscyllium umbratile* (Cephaloscyllium, Scyliorhinidae, Chondrichthyes), belonging the Carcharhiniformes order, is one of the most important aquarium and reef fish, and mainly distribute in the coastwise of China, Vietnam and Japan. Due to small amount, it is regarded as endangered species, and absorbed in red list of International Union for Conservation of Nature (IUCN)^1^. Since the information about *C. umbratile* has been generally scarce, with the development of offshore fishery, increasing research interest has been developed in conservation as well as in scientific and economic topics regarding reef fish^2,3^.

In Chondrichthyes, the typical complete mitochondrial DNA (mtDNA) was circular and approximately 17 kb in length with correspondingly conserved gene content which encoded 37 genes, including 22 transfer RNA (tRNA), 13 protein-coding genes (PCGs), 2 ribosomal RNA (rRNA), a major non-coding control region (D-loop region), and an A + T-rich region^4,5^. Furthermore, genomic information is considered to be reliable for the efficient implementation strategies to study evolutionary relationships, phylogeography and phylogeny^6,7^. Due to its conserved gene content, maternal inheritance, a small genome size, relatively fast evolutionary rate, high copy number and lack of intermolecular genetic recombination^8–10^, mtDNA has been broadly adopted in species identification^11,12^, genome evolution^13–16^ and nonsynonymous (Ka) and synonymous (Ks) substitutions of many species^17–23^.

Moreover, Carcharhiniformes include about 49 genera and over 200 species, and many of them are important economic categories. Nevertheless, several evidences gathered with genome synteny analysis have revealed a number of shared unique mitochondrial gene features in Chondrichthyes, towards a better understanding of the functions and evolution of Chondrichthyes^24–27^. So far, there was still a notably lack of mtDNA information in Carcharhiniformes. In order to provide a theoretical foundation for the conservation strategy of *C. umbratile* within Scyliorhinidae and new sight for further studies of phylogenetically-informative sequence data, in the current study the complete mtDNA of *C. umbratile* was sequenced, assembled and annotated, and compared with other members of Carcharhiniformes.

## Results and Discussion

### Genome size and organization

**Figure 1:**
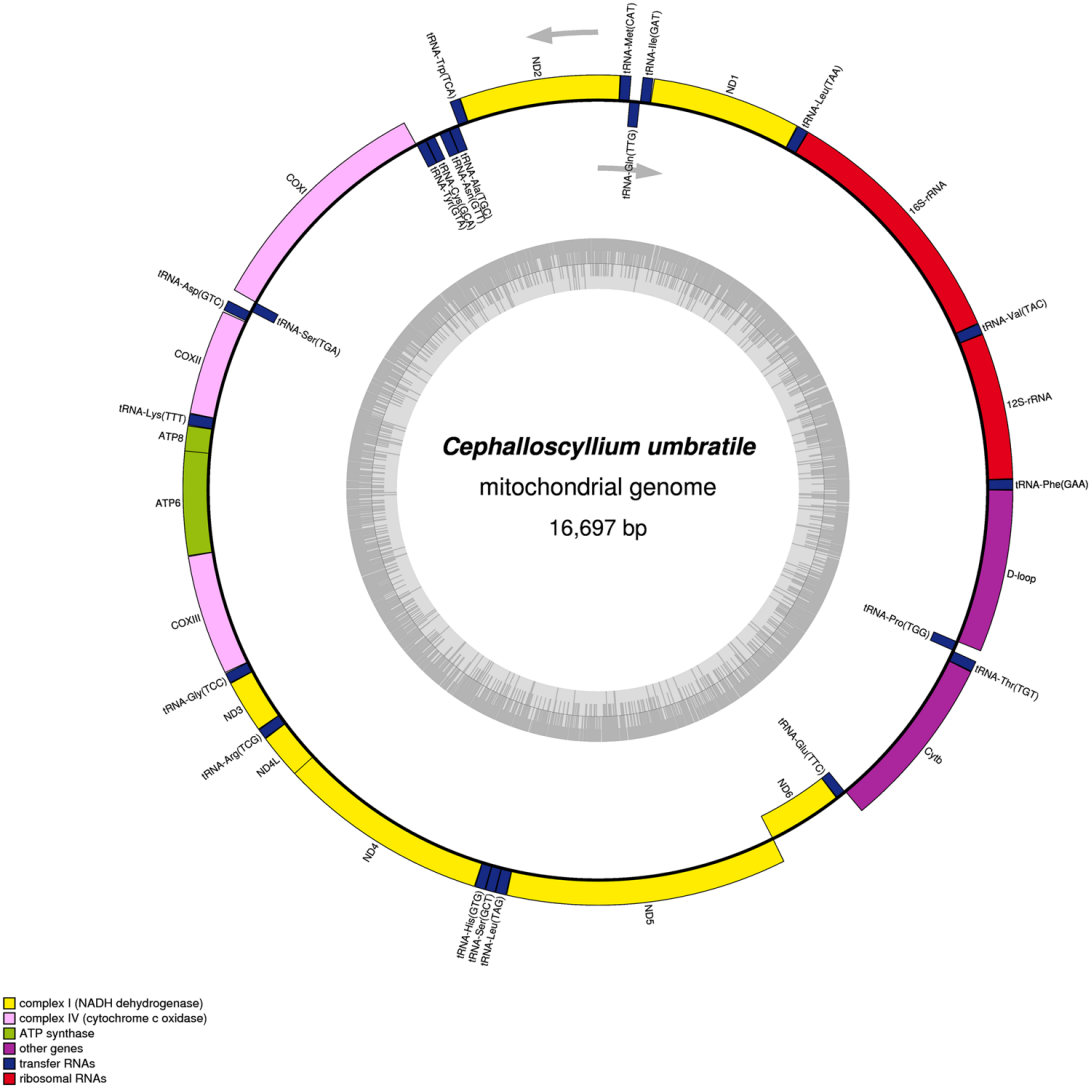
Map of the *Cephalloscyllium umbratile* mitochondrial genome. The genes outside the circle are transcribed clockwise, while the genes inside are transcribed counterclockwise. Gene blocks are filled with different colors as the cutline shows. The inner ring shadow indicates the GC content of the genome.

About 1.5 G raw data is generated with reads length 125 bp. Sequencing coverage and depth (X) of mtDNA data is 100% and approximately 394.23, respectively. Reads number is 52,660 and total bases (bp) is 6,582,500. The mtDNA of *C. umbratile* was a closed-circular DNA molecule of 16,697 bp in length (GenBank: KX354996; Fig. 1, Table 1), which was comparable to other Carcharhiniformes mtDNA ranging from 16,697 bp in *Scyliorhinus canicula* ^25^ to 16,719 bp in *Carcharhinus acronotus* ^28^. Nucleotide BLAST (blastn) of the whole *C. umbratile* mtDNA against other Carcharhiniformes revealed sequence identities with closely related species of 88% (*S. canicula*), 84% (*Proscyllium habereri*), and 84% (*Pseudotriakis microdon*) and with distantly related species of 82% (*Scoliodon laticaudus*), 82% (*Hemigaleus microstoma*), 82% (*Hemipristis elongata*) (Supplementary Table 1). The mtDNA of *C. umbratile* contained 2 rRNA genes, 13 PCGs, 22 tRNA genes and D-loop region. The arrangement of the genes was identical to that of other Scyliorhinidae mtDNA (Table 1)^29,30^. Among these genes, 29 genes (12 PCGs, 2 rRNA genes and 15 tRNA genes) are located on the heavy strand (H-strand) and the others (1 PCGs and 8 tRNA genes) are located on the light strand (L-strand) (Table 1). These obvious features have also been reported in other Carcharhiniformes species^31,32^ and could be regarded as effective markers for authentication at genus and species level.

**Table 1:** Sequence characteristics of *Cephalloscyllium umbratile* mitochondrial genome.

**Locus name**	**One Letter code**	**From**	to	Size	Strand	Nr.of Aminao Acids	Anti-Coden	Inferred Initiation Coden	Inferred Termination Coden	GC_Percent	Intergenic nucleotides*
tRNA-Phe	F	1	69	69	H		GAA			37.68%	0
12S-rRNA		70	1023	954	H					42.98%	0
tRNA-Val	V	1024	1095	72	H		TAC			40.28%	0
16S-rRNA		1096	2764	1669	H					36.01%	0
tRNA-Leu	L	2765	2839	75	H		TAA			44.00%	0
ND1		2840	3814	975	H	324		ATG	TAA	38.97%	3
tRNA-Ile	I	3818	3886	69	H		GAT			39.13%	1
tRNA-Gln	Q	3888	3959	72	L		TTG			29.17%	0
tRNA-Met	M	3960	4029	70	H		CAT			40.00%	0
ND2		4030	5075	1046	H	348		ATG	TA	37.86%	0
tRNA-Trp	W	5076	5144	69	H		TCA			33.33%	1
tRNA-Ala	A	5146	5214	69	L		TGC			31.88%	0
tRNA-Asn	N	5215	5287	73	L		GTT			34.25%	36
tRNA-Cys	C	5324	5389	66	L		GCA			51.52%	1
tRNA-Tyr	Y	5391	5460	70	L		GTA			47.14%	1
COXI		5462	7015	1554	H	517		GTG	TAA	38.61%	0
tRNA-Ser	S	7016	7086	71	L		TGA			45.07%	3
tRNA-Asp	D	7090	7159	70	H		GTC			32.86%	7
COXII		7167	7857	691	H	230		ATG	T	38.35%	0
tRNA-Lys	K	7858	7932	75	H		TTT			44.00%	1
ATP8		7934	8101	168	H	55		ATG	TAA	30.95%	−22
ATP6		8080	8774	695	H	231		ATG	TA	37.55%	0
COXIII		8775	9560	786	H	261		ATG	TAA	42.88%	2
tRNA-Gly	G	9563	9632	70	H		TCC			27.14%	0
ND3		9633	9981	349	H	116		ATG	T	40.97%	0
tRNA-Arg	R	9982	10051	70	H		TCG			32.86%	0
ND4L		10052	10348	297	H	98		ATG	TAA	38.72%	−7
ND4		10342	11722	1381	H	460		ATG	T	37.73%	0
tRNA-His	H	11723	11791	69	H		GTG			18.84%	0
tRNA-Ser	S	11792	11858	67	H		GCT			37.31%	0
tRNA-Leu	L	11859	11930	72	H		TAG			48.61%	0
ND5		11931	13760	1830	H	609		ATG	TAA	35.85%	−4
ND6		13757	14278	522	L	173		ATG	TAA	36.97%	0
tRNA-Glu	E	14279	14348	70	L		TTC			32.86%	2
Cytb		14351	15495	1145	H	381		ATG	TA	39.91%	0
tRNA-Thr	T	15496	15567	72	H		TGT			51.39%	2
tRNA-Pro	P	15570	15638	69	L		TGG			49.28%	0
D-loop		15639	16697	1059	H					31.35%	0

+ and **−** correspond to the H and L strands, respectively.

The nucleotide composition of the mtDNA is biased toward A + T nucleotides (52.9%), which made up of 61.8%, 61.4%, 61.5% and 68.7% in the PCGs, tRNA, rRNA and D-loop region, respectively (Table 2). However, the A + T nucleotide composition in *C. umbratile* was the lowest among Carcharhiniformes. The positive AT skew (0.025) observed here with the presence of more As than Ts, was similar to that only in *Sphyrna tiburo* (0.031), nevertheless, mtDNA in majority of Carcharhiniformes showed negative AT skew (Table 2). The GC skew ranged from −0.324 in *S. tiburo* to 0.040 in *C. macloti* (Table 2). The *C. umbratile* mtDNA was negative (−0.245), indicating the presence of more Cs than Gs.

**Table 2:** Nucleotide composition of the mitochondrial genome in different Carcharhiniformes mtDNA.

Species	Size (bp)	A%	T%	G%	C%	A + T %	AT skewness	GC skewness
**Whole mitogenome**								
*C.umbratile*	16896	27.08	25.78	17.81	29.34	52.86	0.025	−0.245
*S. canicula*	16697	30.80	31.20	14.12	23.87	62.00	−0.006	−0.257
*S. tiburo*	16723	31.26	29.38	13.24	25.94	60.64	0.031	−0.324
*P. habereri*	16708	30.88	31.19	14.18	23.75	62.07	−0.005	−0.252
*C. acronotus*	16719	31.48	30.22	13.18	25.20	61.65	−0.311	0.017
*C.amblyrhynchoides*	16705	31.40	30.34	13.15	25.03	61.79	−0.313	0.020
*C. amboinensis*	16704	31.57	30.42	13.06	24.95	62.00	−0.313	0.019
*C. brevipinna*	16706	31.35	30.13	13.24	25.28	61.47	−0.313	0.020
*C. leucas*	16704	31.47	31.10	13.11	24.32	62.57	−0.300	0.006
*C.longimanus*	16706	31.49	30.01	13.12	25.38	61.50	−0.318	0.024
*C.macloti*	16701	31.61	29.19	13.02	26.18	60.80	−0.336	0.040
*C.melanopterus*	16706	31.28	30.06	13.32	25.33	61.35	−0.311	0.020
*C. plumbeus*	16706	31.25	29.89	13.32	25.54	61.14	−0.314	0.022
*C. sorrah*	16707	31.45	29.60	13.17	25.77	61.05	−0.323	0.030
*L.tephrodes*	16705	31.43	29.77	13.02	25.70	61.25	−0.328	0.027
*L.macrorhinus*	16702	31.71	29.36	13.14	25.80	61.06	−0.325	0.039
*P. microdon*	16700	31.30	32.32	13.63	22.75	63.62	−0.251	−0.016
*T. obesus*	16700	31.38	29.65	13.19	25.78	61.03	−0.323	0.028
**Protein-coding genes**								
*C.umbratile*	11440	28.73	33.02	13.74	24.51	61.75	−0.282	−0.069
*S. canicula*	11430	28.71	33.15	13.85	24.30	61.85	−0.274	−0.072
*S. tiburo*	11430	28.85	31.09	13.06	26.99	59.95	−0.348	−0.037
*P. habereri*	11430	28.83	33.25	13.74	24.18	62.08	−0.275	−0.071
*C. acronotus*	11429	29.44	31.95	12.58	26.02	61.40	−0.348	−0.041
*C.amblyrhynchoides*	11430	29.45	32.30	12.59	25.66	61.75	−0.342	−0.046
*C. amboinensis*	11430	29.58	32.32	12.49	25.61	61.90	−0.344	−0.044
*C. brevipinna*	11430	29.36	31.92	12.65	26.06	61.29	−0.346	−0.042
*C. leucas*	11430	29.43	33.08	12.55	24.94	62.51	−0.331	−0.058
*C.longimanus*	11430	29.55	31.85	12.53	26.07	61.40	−0.351	−0.038
*C.macloti*	11430	29.42	30.83	12.62	27.13	60.25	−0.365	−0.023
*C.melanopterus*	11430	29.32	31.96	12.77	25.93	61.29	−0.340	−0.043
*C. plumbeus*	11430	29.22	31.72	12.81	26.25	60.94	−0.344	−0.041
*C. sorrah*	11430	29.34	31.41	12.74	26.52	60.74	−0.351	−0.034
*L.tephrodes*	11247	29.23	31.52	9.29	26.69	62.80	−0.484	−0.038
*L.macrorhinus*	11430	29.51	30.84	12.69	26.96	60.35	−0.360	−0.022
*P. microdon*	11496	29.51	34.71	13.21	22.57	64.21	−0.262	−0.081
*T. obesus*	11430	29.22	31.35	12.78	26.65	60.57	−0.352	−0.035
**tRNA**								
*C.umbratile*	1538	32.51	28.87	17.43	21.20	61.38	−0.098	0.059
*S. canicula*	1551	31.53	30.82	20.12	17.54	62.35	0.068	0.011
*S. tiburo*	1551	32.62	27.98	17.21	22.18	60.61	−0.126	0.077
*P. habereri*	1553	30.71	29.75	21.31	18.22	60.46	0.078	0.016
*C. acronotus*	1552	30.86	29.70	21.20	30.86	60.57	0.075	0.019
*C.amblyrhynchoides*	1551	32.62	27.92	17.28	32.62	60.54	−0.124	0.078
*C. amboinensis*	1548	32.62	27.78	17.31	32.62	60.40	−0.126	0.080
*C. brevipinna*	1550	30.77	29.55	21.35	30.77	60.32	0.076	0.020
*C. leucas*	1552	0.069	32.73	28.48	17.14	61.21	−0.116	0.069
*C.longimanus*	1553	0.077	32.39	27.75	17.51	60.14	−0.121	0.077
*C.macloti*	1542	0.074	32.49	28.02	17.32	60.51	−0.123	0.074
*C.melanopterus*	1551	0.076	32.43	27.85	17.54	60.28	−0.117	0.076
*C. plumbeus*	1551	0.071	32.17	27.92	17.73	60.09	−0.111	0.071
*C. sorrah*	1552	0.003	27.90	27.71	17.53	58.23	−0.121	0.003
*L.tephrodes*	1551	0.080	32.75	27.92	17.21	60.67	−0.125	0.080
*L.macrorhinus*	1552	31.25	30.15	20.75	17.85	61.4	0.075	0.018
*P. microdon*	1551	31.85	28.76	17.73	21.66	60.61	−0.100	0.051
*T. obesus*	1552	32.73	27.90	17.27	22.10	60.63	−0.123	0.080
**rRNA**								
*C.umbratile*	2623	34.77	26.69	17.69	20.85	61.46	−0.082	0.132
*S. canicula*	2630	34.26	26.50	18.02	21.22	60.76	−0.081	0.128
*S. tiburo*	2623	35.46	26.12	17.35	21.08	61.57	−0.097	0.152
*P. habereri*	2619	35.01	26.42	17.83	20.73	61.44	−0.075	0.140
*C. acronotus*	2629	35.34	26.21	17.15	21.30	61.54	−0.108	0.148
*C.amblyrhynchoides*	2624	35.21	25.88	17.34	21.57	61.09	−0.109	0.153
*C. amboinensis*	2627	35.40	26.19	17.17	21.24	61.59	−0.106	0.150
*C. brevipinna*	2626	35.15	25.89	17.40	21.55	61.04	−0.107	0.152
*C. leucas*	2624	35.18	26.68	17.38	20.77	61.85	−0.089	0.137
*C.longimanus*	2625	35.20	25.71	17.33	21.75	60.91	−0.113	0.156
*C.macloti*	2622	35.28	25.36	17.28	22.08	60.64	−0.122	0.164
*C.melanopterus*	2626	35.03	25.55	17.48	21.93	60.59	−0.113	0.157
*C. plumbeus*	2629	35.26	25.45	17.27	22.02	60.71	−0.121	0.162
*C. sorrah*	2627	35.25	25.58	17.24	21.93	60.83	−0.120	0.159
*L.tephrodes*	2624	35.37	25.69	17.15	21.72	61.10	−0.118	0.159
*L.macrorhinus*	2625	35.73	26.10	17.10	21.07	61.83	−0.104	0.156
*P. microdon*	2624	35.02	26.64	17.72	20.62	61.66	−0.076	0.136
*T. obesus*	2622	35.51	25.55	17.09	21.85	61.06	−0.122	0.163
**Control region**								
*C.umbratile*	1059	34.09	34.56	12.94	18.41	68.65	−0.175	−0.007
*S. canicula*	1051	33.21	33.59	13.23	19.89	66.86	−0.201	−0.006
*S. tiburo*	1087	31.83	32.84	12.60	21.07	65.76	−0.251	−0.016
*P. habereri*	1067	32.61	33.55	13.96	19.87	66.17	−0.175	−0.014
*C. acronotus*	1076	31.69	35.13	13.57	19.61	66.82	−0.182	−0.051
*C.amblyrhynchoides*	1067	31.40	35.05	13.59	19.96	66.45	−0.190	−0.055
*C. amboinensis*	1067	31.68	35.43	13.40	19.49	67.10	−0.185	−0.056
*C. brevipinna*	1068	31.74	35.11	13.67	19.48	66.85	−0.175	−0.050
*C. leucas*	1066	32.27	35.08	13.32	19.32	67.35	−0.184	−0.042
*C.longimanus*	1066	31.24	35.27	13.51	19.98	66.51	−0.193	−0.061
*C.macloti*	1066	33.40	34.80	12.38	19.42	68.20	−0.221	−0.021
*C.melanopterus*	1067	31.58	34.58	13.40	20.43	66.17	−0.208	−0.045
*C. plumbeus*	1063	31.14	35.47	13.55	19.85	66.60	−0.189	−0.065
*C. sorrah*	1066	31.99	34.80	13.23	19.98	66.79	−0.203	−0.042
*L.tephrodes*	1069	32.18	34.89	13.38	19.27	67.26	−0.181	−0.040
*L.macrorhinus*	1063	32.64	34.24	13.26	19.85	66.89	−0.199	−0.024
*P. microdon*	1058	33.74	34.03	11.81	20.42	67.77	−0.267	−0.004
*T. obesus*	1064	31.48	35.53	13.72	19.27	67.01	−0.168	−0.060

Note: The A + T biases of whole mitogenome, protein-coding genes, tRNA, rRNA and control regions were calculated by AT-skew = (A − T)/(A + T) and GC-skew = (G − C)/(G + C), respectively.

### Protein-coding gene features

The PCG region formed 68.5% of the *C. umbratile* mitogenome, and was 11,440 bp long. Furthermore, a contrast of nucleotide composition, AT-skew, and GC-skew of Carcharhiniformes PCGs were exhibited in Table 2. A + T content of the rRNA genes was 61.75%. The AT skew value (−0.282) of the PCG region in the *C. umbratile* mtDNA was higher than that of several reported mtDNA, nevertheless the negative GC skew (−0.069) was similar to that observed in other fish^33,34^.

Each PCG was initiated by a canonical ATN codon, except for *COXI*, which was initiated by a GTG codon (Table 1). Similar results have been documented in other Carcharhiniformes^35,36^. Seven of 13 PCGs (*ND1*, *COXI*, *ATP8*, *COXIII*, *ND4L, ND5*, *ND6*) used a typical TAA termination codon, which was typical for Carcharhiniformes mtDNA^35,36^; whereas *COXII*, *ND3* and *ND4* terminated with a single T and *ATP6*, *ND2* and *Cytb* terminated with TA (Table 1). It was akin to sequenced mtDNA of Carcharhiniformes, including *Triaenodon obesus* ^37^, *Carcharhinus macloti* ^38^, *Mustelus griseus* ^39^, *S. canicula* ^25^ and *C. acronotus* ^28^.

**Figure 2:**
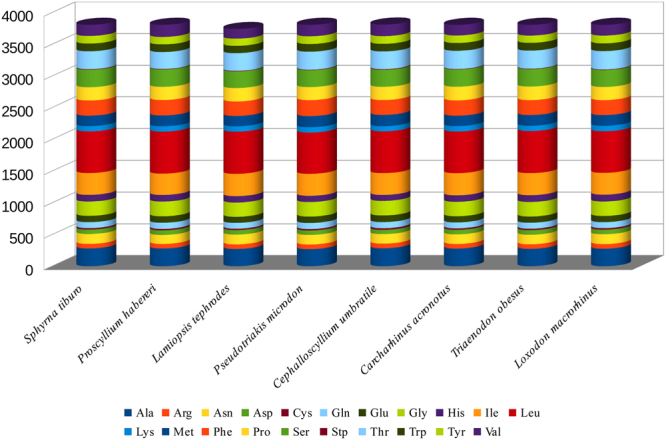
Comparison of codon usage within the mitochondrial genome of members of the Carcharhiniformes. Species *(Sphyrna tiburo, Proscyllium habereri, Lamiopsis tephrodes, Pseudotriakis microdon, Cephalloscyllium umbratile, Carcharhinus acronotus, Triaenodon obesus, Loxodon macrorhinus)* represent the superfamily to which the species belongs (Sphyrna, Proscyllium, Lamiopsis, Pseudotriakis, Cephaloscyllium, Carcharhinus, Triaenodon, Loxodon).

**Figure 3:**
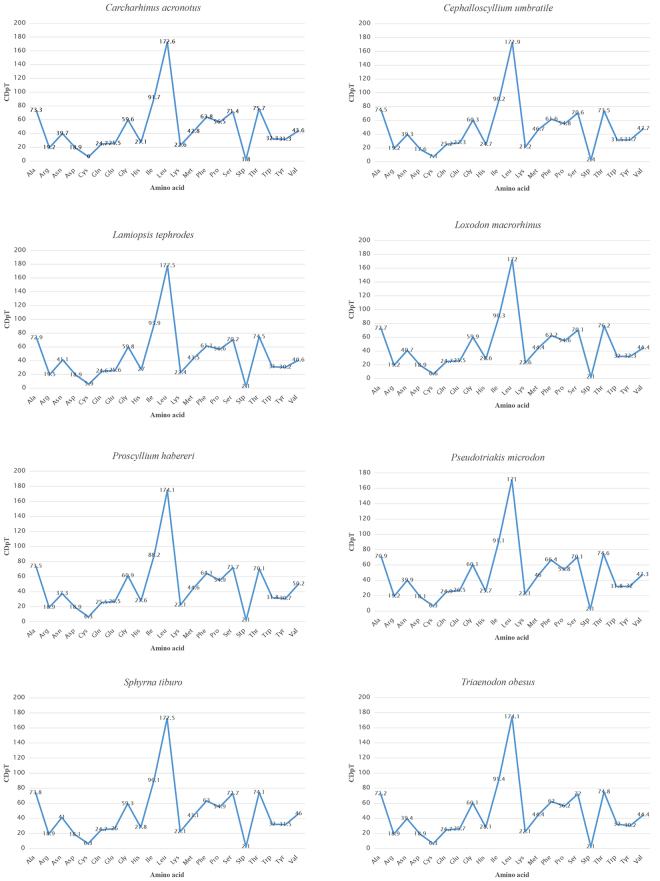
Codon distribution in members of eight superfamilies in the Carcharhiniformes. CDspT = codons per thousand codons.

**Figure 4:**
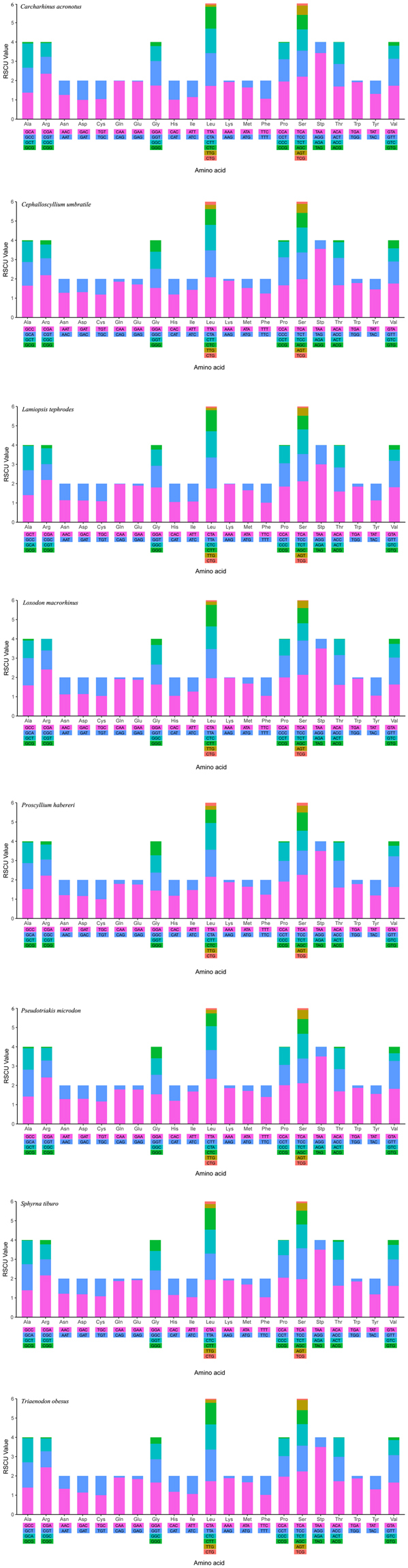
Relative Synonymous Codon Usage (RSCU) of the mitochondrial genome of eight superfamilies in the Carcharhiniformes. Codon families are plotted on the x-axis. Codons indicated above the bar are not present in the mitogenome.

A total of 3,803 amino acids of PCGs are encoded in *C. umbratile*. In addition, the codon usage is shown in Table 3. The most frequent amino acids in the PCGs of *C. umbratile* were Leucine (17.3%), Isoleucine (9.02%) and Alanine (7.45%) (Table 3). Relative synonymous codon usage (RSCU) analysis of PCGs in *C. umbratile* revealed that the codons encoding Leu, Thr, Ala, Arg, Gln, Gly, Pro and Ser were the most frequently present, nevertheless those encoding Asn, Asp, Cys and Lys were rare (Fig. 2). In the PCGs of the eight species examined, codon distributions and amino acid content were corresponding among species (Fig. 3). It was declared that conserved amino acid sequences were present among those fish^28,32,40^. Moreover, codons with A or T in the third position were overused in comparison to other synonymous codons, for example, the codons for glutamine CAG and GAG were rare, while the synonymous codons CAA and GAA were prevalent (Fig. 4), which is consistent with previous observations of Carcharhiniformes^36^.

**Table 3:** Codon usage of *Cephalloscyllium umbratile* mitochondrial protein-coding genes.

Amino acid	Codon	Number	Frequency (%)	RSCU	Amino acid	Codon	Number	Frequency (%)	RSCU
Ala	GCC	117	3.07	1.65		CAC	56	1.47	1.19
	GCA	87	2.28	1.23		CAT	38	0.99	0.81
	GCT	76	1.99	1.07	Ile	ATT	246	6.45	1.43
	GCG	4	0.11	0.06		ATC	98	2.57	0.57
Arg	CGA	40	1.05	2.19	Leu	TTA	229	6.01	2.08
	CGT	16	0.42	0.88		CTA	153	4.02	1.39
	CGC	13	0.34	0.71		CTT	145	3.81	1.32
	CGG	4	0.10	0.22		CTC	90	2.36	0.82
Asn	AAT	96	2.52	1.28		TTG	24	0.63	0.22
	AAC	54	1.42	0.72		CTG	18	0.47	0.16
Asp	GAT	44	1.15	1.31	Lys	AAA	77	2.02	1.90
	GAC	23	0.60	0.69		AAG	4	0.10	0.10
Cys	TGT	16	0.42	1.19	Met	ATA	136	3.57	1.53
	TGC	11	0.29	0.81		ATG	42	1.10	0.47
Gln	CAA	89	2.34	1.85	Phe	TTT	146	3.83	1.24
	CAG	7	0.18	0.15		TTC	89	2.34	0.76
	GAA	89	2.34	1.71	Pro	CCA	87	2.28	1.67
	GAG	15	0.39	0.29		CCC	76	1.99	1.45
Gly	GGA	88	2.31	1.53		CCT	42	1.10	0.80
	GGC	57	1.50	0.99		CCG	4	0.10	0.08
	GGT	51	1.34	0.89	Ser	TCA	89	2.34	1.99
	GGG	34	0.89	0.59		TCT	62	1.63	1.38
His	CAC	56	1.47	1.19		TCC	58	1.52	1.29
Amino acid	Codon	Number	Frequency (%)	RSCU	Amino acid	Codon	Number	Frequency (%)	RSCU
	AGC	34	0.89	0.76		ACG	7	0.18	0.1
	AGT	21	0.55	0.47	Trp	TGA	107	2.81	1.78
	TCG	5	0.13	0.11		TGG	13	0.34	0.22
Stp*	TAA	7	0.18	4	Tyr	TAT	88	2.31	1.45
	AGA	0	0	0		TAC	33	0.87	0.55
	AGG	0	0	0	Val	GTA	80	2.10	1.76
	TAG	0	0	0		GTT	52	1.36	1.14
Thr	ACA	117	3.07	1.67		GTC	31	0.81	0.68
	ACC	99	2.60	1.41		GTG	19	0.50	0.42
	ACT	57	1.50	0.81

### Transfer RNAs and ribosomal RNAs

The representative complement structures of 22 tRNAs were identified in the *C. umbratile* mtDNA, ranging from 62 bp (tRNA^Thr^) to 76 bp (tRNA^Lys^)^35,36^ for 1,538 bp in total (Table 1). Of those, the highest A + T content of tRNAs was *S. canicula* and the lowest was *C. sorrah*. Fifteen tRNA genes were encoded on the H strand while the remains were located in the L strand (Table 1). The overall A+T content of tRNAs was 61.38% which was approximate to that observed in *Loxodon macrorhinus* (61.4%). The negative AT skew (−0.098) and positive GC skew (0.059) showed in the *C. umbratile* mtDNA were also analogous with several sequenced Carcharhiniformes (Table 2).

**Figure 5:**
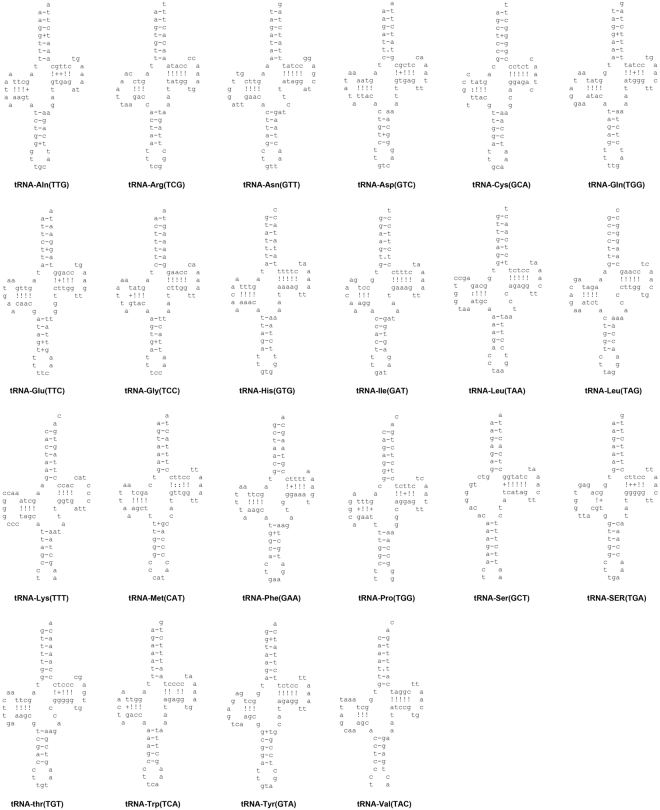
Putative secondary structures for 22 tRNA genes in mitochondrial genome of *Cephalloscyllium umbratile*. Watson-Crick and GT bonds are illustrated by “−” and “+”, respectively.

The forecasted tRNAs were shown in Fig. 5. All of the tRNAs could be folded into classic clover-leaf secondary structures in *C. umbratile*, except for tRNA-Ser (GCT), which lacked the dihydrouridine ‘DHU’ arm (Fig. 5). The ‘DHU’ arm of this tRNA was a large loop instead of the conserved stem-and-loop structure. Due to a representative characteristics^41^, it was also observed in other Chondrichthyes mtDNA, including *Chiloscyllium griseum* ^42^ *T. obesus* ^37^ and so on. Fifteen of the tRNA genes were each observed to have at least one G-T mismatches in their respective secondary structures, which forming a weak bond. Five T-T mismatches were present in the respective amino acid acceptor stems of *tRNA* ^*Asp*(GTC)^, *tRNA* ^*Cys*(GCA)^, *tRNA* ^*His*(GTG)^, *tRNA* ^*Ile*(GAT)^ and tRNA^*Met*(CAT)^ (Fig. 5). Interestingly, A-G mismatch was also present in tRNA-Leu (TAA). Unmatched base pairs perceived in tRNA sequences can be amended by RNA-editing mechanisms that were well known for vertebrate mtDNA^43^.

The A + T content of the rRNA genes was 61.46%, indicating an A+C-rich trend as in other Scyliorhinidae fish^25^. AT and GC skews were negative (−0.082) and positive (0.132), respectively (Table 2). The *12S rRNA* and *16S rRNA* subunit gene of *C. umbratile* was 954 bp and 1,668 bp in length, respectively. As in other vertebrates^44^, both two genes are separated by the *tRNA* ^*Val*^ gene, and located between *tRNA* ^*Phe*^ and *tRNA* ^*Leu*(UUR)^ (Fig. 1, Table 1). The overall content of the rRNA was analogous to that observed for other Carcharhiniformes.

### The control region

The length of D-loop region of *C. umbratile* was 1,059 bp, which was less long than majority of Carcharhiniformes. The A + T content was 68.65%, and equal with other Carcharhiniformes (Table 2), which was consistent with the findings of previous reports on other teleosts^33,45,46^. Moreover, both of the AT-skew and GC-skew were strongly negative (Table 2).

### Overlapping and intergenic spacer regions

There were three gene boundaries where bases overlapped between adjacent genes, ranging from 4–22 bp in size. The longest overlapping region was 22 bp between *ATP8* and *ATP6* (Table 1) which has been documented in several other Chondrichthyes mtDNA^4,25,32^. Moreover, intergenic spacers of *C. umbratile* were spread over 12 locations and ranged from 1–36 bp, making up 60 bp in total, and the longest intergenic spacer region (36 bp) was between *tRNA* ^*Asn*^ and *tRNA* ^*Cys*^ (Table 1).

### Synonymous and nonsynonymous substitutions

**Figure 6:**
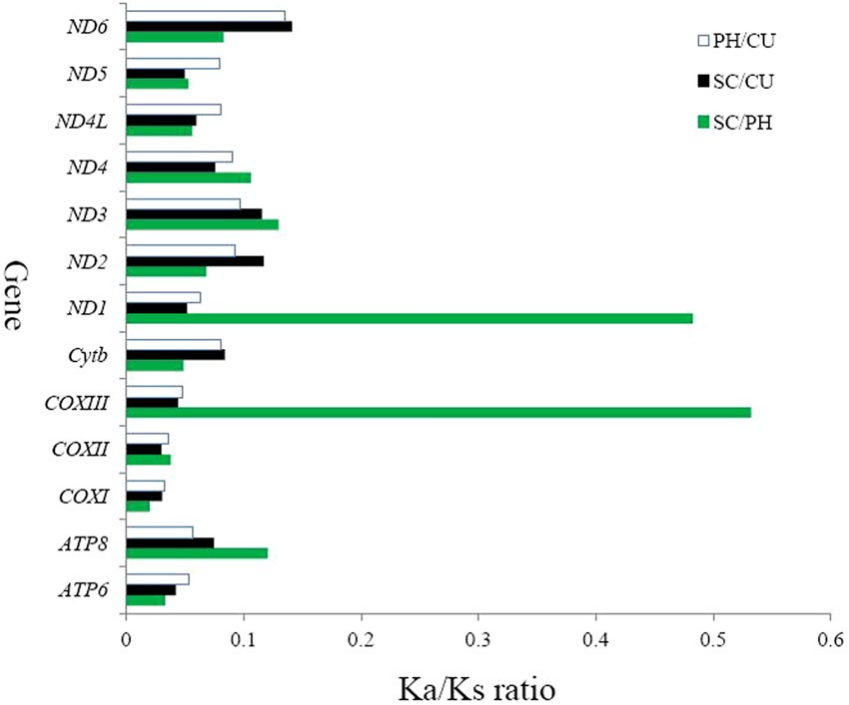
Ka/Ks ratios for the 13 mitochondrial protein-coding genes among the reference *Cephalloscyllium umbratile* (CU), *Scyliorhinus canicula* (SC), *Proscyllium habereri* (PH).

The ratio of Ka/Ks is generally regarded as a pointer of selective pressure and evolutionary relations at the molecular level among homogenous or heterogeneous species^47,48^. It is reported that Ka/Ks > 1, Ka/Ks = 1, and Ka/Ks < 1 popularly declared positive selection, neutral mutation and negative selection, respectively^49^. To investigate the evolutionary rate differences in three Carcharhiniformes mtDNA (*C. umbratile, S. canicula* and *P. habereri*), sequence divergences by counting Ka and Ks substitution rates were next calculated. The Ka/Ks values of 13 PCGs varied from 0.0198 (*COXI*) to 0.5322 (*ATP8*) and were less than 0.6 (Ka was lower than Ks) for all other genes which indicated a strong purifying and negative selection in those fishes (Fig. 6). Our result of the Ka/Ks ratio illustrated that the multitudinous genes evolved under strong negative selection which meant natural selection against profitless mutations with negative selective coefficients^50^. The percentages of variable sites of SC/PH were the highest in *COXIII* and *ND1* among the groups, while the percentages was the least in *COXI* gene, which indicated that *COXIII* and *ND1* were under the least selective pressure, and *COXI* was under the most selective pressure among all mitochondrial proteins. In *C. umbratile* and *S. canicula*, the ratio of Ka/Ks was the least in all 13 protein-coding genes compared to *P. habereri*, implying that these two Scyliorhinidae fish had the closer phylogenetic relationship than *P. habereri*, which was consistent with their rozmieszczenie naturalne and ecological habit^25^.

### Phylogeny

**Figure 7:**
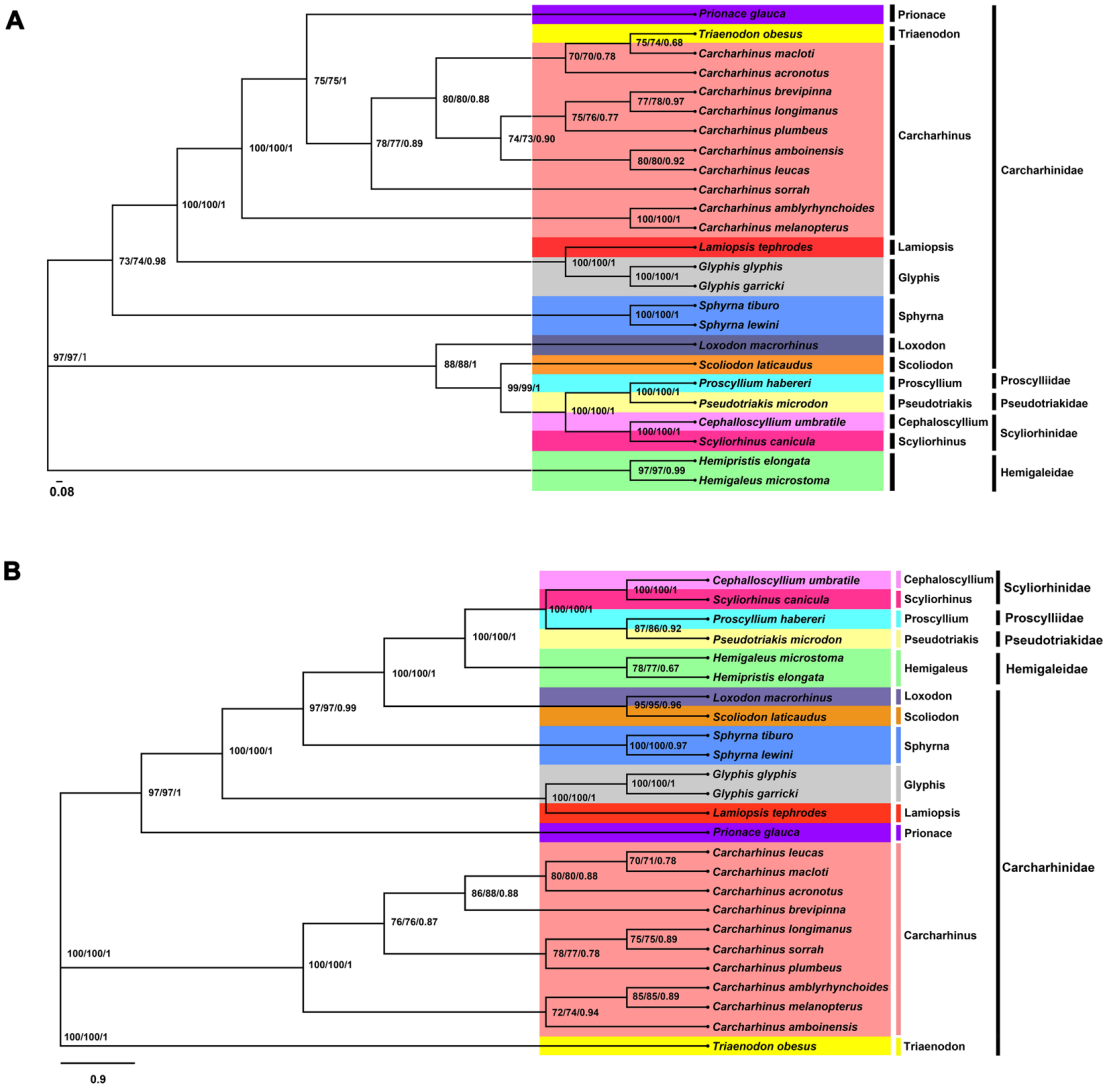
Phylogenetic trees of *Cephalloscyllium umbratile* relationships from the nucleotide (**A**) and amino acid datasets (**B**). Sequences alignment of mtDNA were analyzed using the MEGA 6.0 and Phylobayes 3.3 f software with Maximum likelihood (ML), Maximum parsimony (MP) and Bayesian inference (BI) method, respectively. The accession numbers of the sequences used in the phylogenetic analysis are listed in Supplementary Table 1.

To understand the phylogenetic relationships among Carcharhiniformes, base on Maximum Likelihood (ML), Neighbor Joining (NJ) and Bayesian Inference (BI) methods, a dataset of 25 species containing the concatenated nucleic acid and amino acid sequences of 13 PCGs was used to generate phylogenetic relationships (Fig. 7). The topologies of the 6 phylogenetic trees were analogical in our study. The results implied that strong statistics supported for the following relationship among the 5 Superfamily (Scyliorhinidae, Carcharhinidae, Hemigaleidae, Proscylliidae, Pseudotriakidae) (Fig. 7A,B). This clustered pattern of 5 Superfamily was broadly consistent with previous studies^32,42,51–53^. Furthermore, based on all of ML, NJ and BI methods, 5 superfamily divided into 13 closely genera, and *C. umbratile* (Cephaloscyllium) was most closely related to *S. canicula* (Scyliorhinus) in Scyliorhinidae, which was accord with the tendency of nucleotide sequence identity and a recent study^51,54–57^. Scyliorhinidae was most closely related to Proscylliidae. Additionally, further taxon sampling within Scyliorhinidae and related superfamilies is required to resolve the location of Scyliorhinidae in Carcharhiniformes.

## Materials and Methods

### Sample collection and mitochondrial DNA extraction

*C. umbratile* juveniles were collected from South China Sea (Longitude 5°20.267′ N and latitude 109°48.435′ E) in September 2014 and directly frozen. Muscle tissues were used for DNA extraction according to the Genomic DNA Extraction Kit’s instructions (TaKaRa MiniBEST Universal Genomic DNA Extraction Kit Ver.5.0, Japan). The quantity (concentration) of isolated total DNA was determined by NANODROP 2000 spectrophotometer (Thermo Scientific, USA). Furthermore, quality of extracted DNA was assessed by electrophoresis on a 1% agarose gel stained with Gel Red™ (Biotium).

### Genome sequencing

According to NEBNext DNA sample libraries kit (NEB, New England)‘s instructions, the normalized DNA (4 μg) was used to structure the paired-end library. Size and quantification estimation of the library were implemented by a Bioanalyzer 2100 High Sensitivity DNA chip (Agilent, USA). Illumina HiSeq. 2500 (2 × 101 bp paired-end reads) (Illumina, USA) was used to sequence the normalized library (2 nM).

### Genome assembly and annotation

A de novo assembly of the paired-end HiSeq reads was performed using SeqMan NGen (http://www.dnastar.com/t-tutorials-seqman-ngen.aspx) (DNASTAR Inc., Madison, WI, USA)^58^. Assembly parameters minimum match percentage, match spacing, match size, gap penalty, mismatch penalty, maximum gap length and expected genome length were set to 93, 10, 50, 30, 20, 6% and 16,000, respectively. Accordance sequence was exported and ends were manually edited to remove duplicated nucleotides. Subsequently, the assembled sequences were aligned to NCBI nt database with blastn method (https://blast.ncbi.nlm.nih.gov/). Sequences that mapping to Carcharhiniformes mtDNA were considered as *C. umbratile* mtDNA. To verify the accuracy of the assembled mtDNA sequence, the primers (Supplementary Table 2) were used to amplify the genome sequence. The procedure of PCR amplification was referred from Sun *et al*.^59^. To determine whether this method was accurate, the sequence segments of same genomic region obtained from Sanger sequencing and shotgun assembly were compared. If they were identical, that meaning this method was precise. Moreover, the PCGs, rRNA genes, tRNA genes and D-loop region of mtDNA were annotated by MitoAnnotator (http://mitofish.aori.u-tokyo.ac.jp/annotation/input.html)^60^ with parameters of complete circular genome. The mtDNA sequence of *C. umbratile* has been deposited in the GenBank database under accession numbers KX354996.

### Genome sequence analysis

tRNAscan-SE Search Server 1.21 program was used to primordially determine Transfer RNAs^61,62^. The gene map of *C. umbratile* mtDNA was built by OGDRAW1.2 and embellished manually^63^. The strand skew values were reckoned in terms of the formulae by Perna and Kocher (1995)^64^. The mode of “models- > Compute Codon Usage Bias” was chose to obtain RSCU in MEGA 6.0^65^. To determine the evolutionary branching of the Carcharhiniformes lineage, codon usage in the 13 PCGs and the rates of Ka/Ks substitutions in the mtDNA of Carcharhiniformes were calculated by DnaSP 5.10.01^66^. To describe base composition, we analyzed skew as described as below: AT-skew = (A − T)/(A + T) and GC-skew = (G − C)/(G+C)^67^.

### Phylogenetic analysis

To discuss the phylogenetic position of Carcharhiniformes, a total of 25 species of 13 PCG sequences were used to perform phylogenetic analysis, including those of *C. umbratile*. Alignments of the 13 concatenated PCGs nucleotide and amino acid sequences were conducted using ClustalX version 2.0 with default parameters^68^. Phylogenetic analyses for each concatenated dataset was performed using ML, MP and BI methods with MEGA 6.0 and Phylobayes 3.3 f, respectively^65,69^. The methods of ML and MP analysis were performed with GTR+I+G model and Subtree-Purning-Regrafting (SPR) model using MEGA 6.0, respectively. The evaluation of node accuracy was done by using 1,000 bootstrap replicates in MEGA 6.0 with default parameters. Furthermore, BI analysis was selecting the CAT-GTR model, two independent Markov chain Monte Carlo (MCMC) chains were run for 10,000 cycles. The phylogenetic tree was embellished using FigTree v1.4.2 (http://tree.bio.ed.ac.uk/software/figtree/).

## Electronic supplementary material

Supplementary Information
