# Supplementary material for: Sequencing and characterization of the complete mitochondrial genome of Japanese Swellshark (Cephalloscyllium umbratile)
Source: Sci Rep. 2017 Nov 10;7:15299. doi: 10.1038/s41598-017-15702-0 (PMC5681689; doi:10.1038/s41598-017-15702-0)
Supplement: Supplementary file 1 — Supplementary Information [file 41598_2017_15702_MOESM1_ESM.pdf]

Title page

Sequencing and characterization of the complete mitochondrial genome of Japanese Swellshark (*Cephalloscyllium umbratile*)

Ke-Cheng Zhu [1,2,3], Yin-Yin Liang [1], Na Wu [1], Hua-Yang Guo [1,2], Nan Zhang [1,2], Shi-Gui Jiang [1,2,4] & Dian-Chang Zhang [1,2,3 *]

[1]Key Laboratory of South China Sea Fishery Resources Exploitation and Utilization, Ministry of Agriculture, South China Sea Fisheries Research Institute, Chinese Academy of Fishery Sciences, 231 Xingang Road West, Haizhu District, Guangzhou 510300, China;

[2]Engineer Technology Research Center of Marine Biological Seed of Guangdong Province, Guangzhou, Guangdong Province, The People's Republic of China;

[3]Key Laboratory of Fishery Ecology & Environment, Guangdong Province;

[4]South China Sea Bio-Resource Exploitation and Utilization Collaborative Innovation Center.

*Corresponding author:

Dr. Dian C. Zhang

231 Xingang Road West, Haizhu District, Guangzhou City, Guangdong 510300, PR China.

E-mail address: zhangdch@scsfri.ac.cn

Tel.: +86 02089108316; fax: +86 02089022702

Supplementary table 1

Superfamily	Genera	Species	Size (bp)	Genbank No.	Identity (%)
Scyliorhinidae	Cephaloscyllium	*Cephalloscyllium umbratile*	16896	KX354996	100
	Scyliorhinus	*Scyliorhinus canicula*	16697	Y16067.1	88
Proscylliidae	Proscyllium	*Proscyllium habereri*	16708	NC030216	84
Pseudotriakidae	Pseudotriakis	*Pseudotriakis microdon*	16700	NC022735	84
Carcharhinidae	Sphyrna	*Sphyrna tiburo*	16723	KM453976	83
		Sphyrna lewini	16726	NC022679	83
	Carcharhinus	*Carcharhinus acronotus*	16719	NC024055	83
		Carcharhinus amblyrhynchoides	16705	NC023948	83
		Carcharhinus amboinensis	16704	NC026696	83
		Carcharhinus brevipinna	16706	NC027081	83
		Carcharhinus leucas	16704	NC023522	83
		Carcharhinus longimanus	16706	NC025520	83
		Carcharhinus macloti	16701	NC024862	83
		Carcharhinus melanopterus	16706	NC024284	83
		Carcharhinus plumbeus	16706	NC024596	83
		Carcharhinus sorrah	16707	NC023521	83
	Triaenodon	*Triaenodon obesus*	16700	NC026287	83
	Glyphis	*Glyphis glyphis*	16701	NC021768	83
		Glyphis garricki	16702	KF646786	83
	Prionace	*Prionace glauca*	16705	NC022819	83
	Lamiopsis	*Lamiopsis tephrodes*	16705	KT698047	83
	Loxodon	*Loxodon macrorhinus*	16702	NC029843	83
	Scoliodon	*Scoliodon laticaudus*	16695	KP336547	82
Hemigaleidae	Hemigaleus	*Hemigaleus microstoma*	16701	NC029400	82
		Hemipristis elongata	16691	KU508621	82

Supplementary table 2

Primers used to verify the accuracy of the assembled mitochondrial genome sequence.

Name	Primer sequences (5'–3')	Product length (bp)	Tm (℃)
CuF1	ATGGCACTGAAGATGCTAAGATG	5126	53
CuR1	AGCTTTGAAGGCTTTTGGTCT		
CuF2	CACTTTGATAGAGTGGATAATGA	4945	53
CuR2	ATGTGCTTGGTGAGCCATTATAC		
CuF3	TGAAATTGACCATGATTATAAGCT	4992	52
CuR3	AATGGTATACCTGTGAGGGCTA		
CuF4	CTTGGTGCAACTCCAAGCAAG	5064	57
CuR4	TCGTATAACCGCGGTGGCTGGCA		
